# Ventricular structure in ARVC: going beyond volumes as a measure of risk

**DOI:** 10.1186/s12968-016-0291-9

**Published:** 2016-10-14

**Authors:** Kristin McLeod, Samuel Wall, Ida Skrinde Leren, Jørg Saberniak, Kristina Hermann Haugaa

**Affiliations:** 1 Cardiac Modelling Department, Simula Research Laboratory, PO Box 134, Oslo, Norway; 2 Department of Cardiology and Institute for Surgical Research, Oslo University Hospital, Rikshospitalet, Oslo, Norway; 3 University of Oslo, Oslo, Norway; 4 Center for Cardiological Innovation, Oslo, Norway

**Keywords:** ARVC, CMR, Principal component analysis, Shape modes, Risk assessment

## Abstract

**Background:**

Altered right ventricular structure is an important feature of Arrhythmogenic Right Ventricular Cardiomyopathy (ARVC), but is challenging to quantify objectively. The aim of this study was to go beyond ventricular volumes and diameters and to explore if the shape of the right and left ventricles could be assessed and related to clinical measures. We used quantifiable computational methods to automatically identify and analyse malformations in ARVC patients from Cardiovascular Magnetic Resonance (CMR) images. Furthermore, we investigated how automatically extracted structural features were related to arrhythmic events.

**Methods:**

A retrospective cross-sectional feasibility study was performed on CMR short axis cine images of 27 ARVC patients and 21 ageing asymptomatic control subjects. All images were segmented at the end-diastolic (ED) and end-systolic (ES) phases of the cardiac cycle to create three-dimensional (3D) bi-ventricle shape models for each subject. The most common components to single- and bi-ventricular shape in the ARVC population were identified and compared to those obtained from the control group. The correlations were calculated between identified ARVC shapes and parameters from the 2010 Task Force Criteria, in addition to clinical outcomes such as ventricular arrhythmias.

**Results:**

Bi-ventricle shape for the ARVC population showed, as ordered by prevalence with the percent of total variance in the population explained by each shape: global dilation/shrinking of both ventricles (44 %), elongation/shortening at the right ventricle (RV) outflow tract (15 %), tilting at the septum (10 %), shortening/lengthening of both ventricles (7 %), and bulging/shortening at both the RV inflow and outflow (5 %). Bi-ventricle shapes were significantly correlated to several clinical diagnostic parameters and outcomes, including (but not limited to) correlations between global dilation and electrocardiography (ECG) major criteria (*p = 0.002*), and base-to-apex lengthening and history of arrhythmias (*p = 0.003*). Classification of ARVC vs. control using shape modes yielded high sensitivity (96 %) and moderate specificity (81 %).

**Conclusion:**

We presented for the first time an automatic method for quantifying and analysing ventricular shapes in ARVC patients from CMR images. Specific ventricular shape features were highly correlated with diagnostic indices in ARVC patients and yielded high classification sensitivity. Ventricular shape analysis may be a novel approach to classify ARVC disease, and may be used in diagnosis and in risk stratification for ventricular arrhythmias.

**Electronic supplementary material:**

The online version of this article (doi:10.1186/s12968-016-0291-9) contains supplementary material, which is available to authorized users.

## Background

Arrhythmogenic right ventricular cardiomyopathy (ARVC) is an inherited cardiomyopathy affecting approximately 1 in 5000 individuals [1]. The disease is characterized by desmosomal dysfunction, leading to cell necrosis and fibro-fatty replacement of the myocardium, with disease generally starting in the right ventricle. Desmosomal dysfunction leads to changes in heart structure and function also affecting the electrical propagation through the ventricles, which may cause life-threatening ventricular arrhythmias.

Diagnosis and treatment is relatively straightforward in severe cases of definite ARVC. However, in mild-to-moderate cases diagnosis is challenging due to the complex nature of the disease, and treatment planning is made on a case-by-case basis. Diagnosis of ARVC is currently guided by the Task Force criteria (TFC), revised in 2010 [2]. Cardiovascular Magnetic Resonance (CMR) is commonly used for diagnostic purposes in ARVC patients to identify global and regional structural abnormalities, dysfunction and for identification of fatty/fibrotic regions [3, 4]. Although regional structural abnormalities at the inflow tract, outflow tract and apex of the RV are known in these patients [5], quantitative measures of structural abnormalities are generally limited to global measures including end-diastolic volume (EDV), end-systolic volume (ESV) or ejection fraction (EF) by CMR, in addition to right ventricular outflow tract (RVOT) diameter from echocardiography. Though techniques for imaging and assessment of the entire RV are being developed using 3D echo [6], echocardiographic assessment of ARVC has typically been limited to 2D images, giving information for only a small part of the RV. Structural abnormalities can be qualitatively analysed from images by visual assessment. However, comparing qualitative measures from different observers and different patients is challenging and has several limitations [5].

Descriptive three-dimensional (3D) measures can give more information in terms of localisation and size of an abnormality, e.g. bulging or aneurysms. In terms of computational methods, the normal structure (shape) can be described by the mean (average) shape in the population and the degree of variation (i.e. the variance) can be described by the modes (shapes) around the mean. This task was recently addressed for the left ventricle (LV) using a mean shape model and deforming the mean to each subject by assigning landmarks, such as the base, apex and junctions between the ventricles, to personalise the shape and to correlate with clinical measures in atherosclerosis patients [7]. A similar approach using principal component analysis (PCA) [8], but without requiring landmarks to compute the shape modes, was used to correlate right ventricle (RV) shapes with clinical measures in Tetralogy of Fallot patients [9].

ARVC diagnosis, monitoring of disease progression and patient risk stratification are currently limited by examination techniques, heterogeneous structural disease progression and reliable markers of risk for life threatening events [10–13]. In this work we sought to go beyond ventricular volumes and ejection fraction as measures of structural abnormalities by using quantifiable computational methods to automatically identify and analyse malformations in ARVC patients compared to control subjects following the method described in [14]. Furthermore, we aimed to investigate if specific structural shapes were related to clinical parameters and ventricular arrhythmias in ARVC. We hypothesized that these specific shape features, quantified using modern computational approaches, would be associated with clinical outcome and could give additional information on the disease.

## Methods

### Study population

We included 27 ARVC patients from the Unit of Genetic Cardiac Diseases, Oslo University Hospital, Rikshospitalet, Oslo, Norway. ARVC was diagnosed according to the current TFC [2] defined as definite ARVC (2 major criteria, 1 major and 2 minor criteria, or 4 minor TFC diagnostic criteria from different categories), borderline ARVC (1 major and 1 minor or 3 minor TFC diagnostic criteria) and possible ARVC (1 major or 2 minor TFC diagnostic criteria). ARVC patients with short-axis CMR available at the time of the study (2015) and scanned on a machine from the same vendor (Siemens) and with the same magnet strength (1.5 T) were chosen for the study. In addition, 21 asymptomatic subjects were recruited as ageing controls to compare the difference between age-related and disease-related remodelling. These controls were taken from a cohort of patients referred with suspected ischemic cardiac disease, but with normal angiography excluding coronary artery disease and with normal volumes and EF by echocardiography and by CMR. The control subjects were randomly chosen, with no prior evaluation of the images to avoid bias in subject selection.

Parameters from the TFC 2010 were collected from the medical records, including electrocardiography (ECG) findings (depolarization and repolarization abnormalities from resting ECG and analyses of signal-averaged ECG), structural and functional alterations by echocardiography and CMR, genetic findings and clinical outcome. Echocardiography was performed as previously described in [15] and parameters for ARVC diagnosis by TFC were analysed. Ventricular arrhythmias were defined as documented non-sustained or sustained ventricular tachycardia by Holter, exercise test or ICD recordings. Also, syncope of assumed cardiac origin and aborted cardiac arrests were recorded. BSA was computed for each patient using the Dubois formula: BSA(*m* ^*2*^) = 0.007184 × *weight(kg)* ^0.425^ × *height(cm)* ^0.725^.

### Cardiovascular magnetic resonance

In order to analyse ARVC-specific structural abnormalities in comparison to normal ventricular structural patterns, CMR-based datasets of ARVC patients and control subjects were collected from retrospective databases and pooled for analysis. All participants had the CMR performed with a Siemens 1.5 T scanner, and gave written informed consent. Short-axis CMR was acquired for each subject, fully covering both ventricles, with voxel size between 1.33 *mm* and 1.65 *mm* and slice thickness of 6 *mm*.

### Mean anatomical model construction

Automatic techniques to detect heart wall boundaries, based on image contrast between tissue and the blood pool, were applied first. User interaction was then required to visually check that the algorithms gave good matching between the contours and the images. If segmentation errors were observed (i.e. if the image segmentation method was unable to correctly identify the borders), correction of the surface models was performed manually. All segmentation was performed with the Segment software, MedViso (http://www.medviso.com/) [16] by a single user, prior to any statistical analysis.

**Fig. 1:**
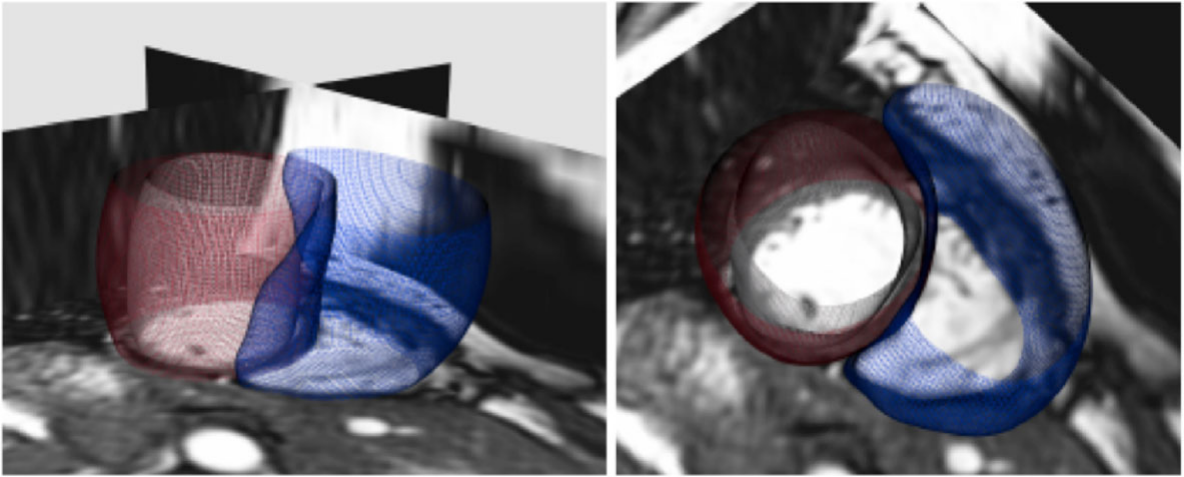
Two views of a short-axis CMR with the subject-specific 3D bi-ventricle model overlaid. The LV endocardium is shown in white, the LV epicardium is shown in red, and the RV endocardium in blue

Fully automatic methods for pre-processing the data from the segmentation were developed to enable population-wide comparison and analysis of the structure. The anatomical models were pre-processed to firstly correct for the slice misalignment caused by breath holds, then to eliminate differences in orientation and pose due to the different image locations, and finally to account for the different temporal sampling of each image sequence. Once all subjects were aligned to a common space with a common temporal sampling, the ED and ES phases were identified and the mean shapes for each time instance were computed individually on each surface model (the LV endocardium, LV epicardium and RV endocardium see Fig. 1). Note that the RV epicardium was not included given the thin wall of the RV, and resulting difficulties in delineating these from CMR images. See Additional file 1 for a full description of the methods used to obtain 3D models from the CMR images.

**Fig. 2:**
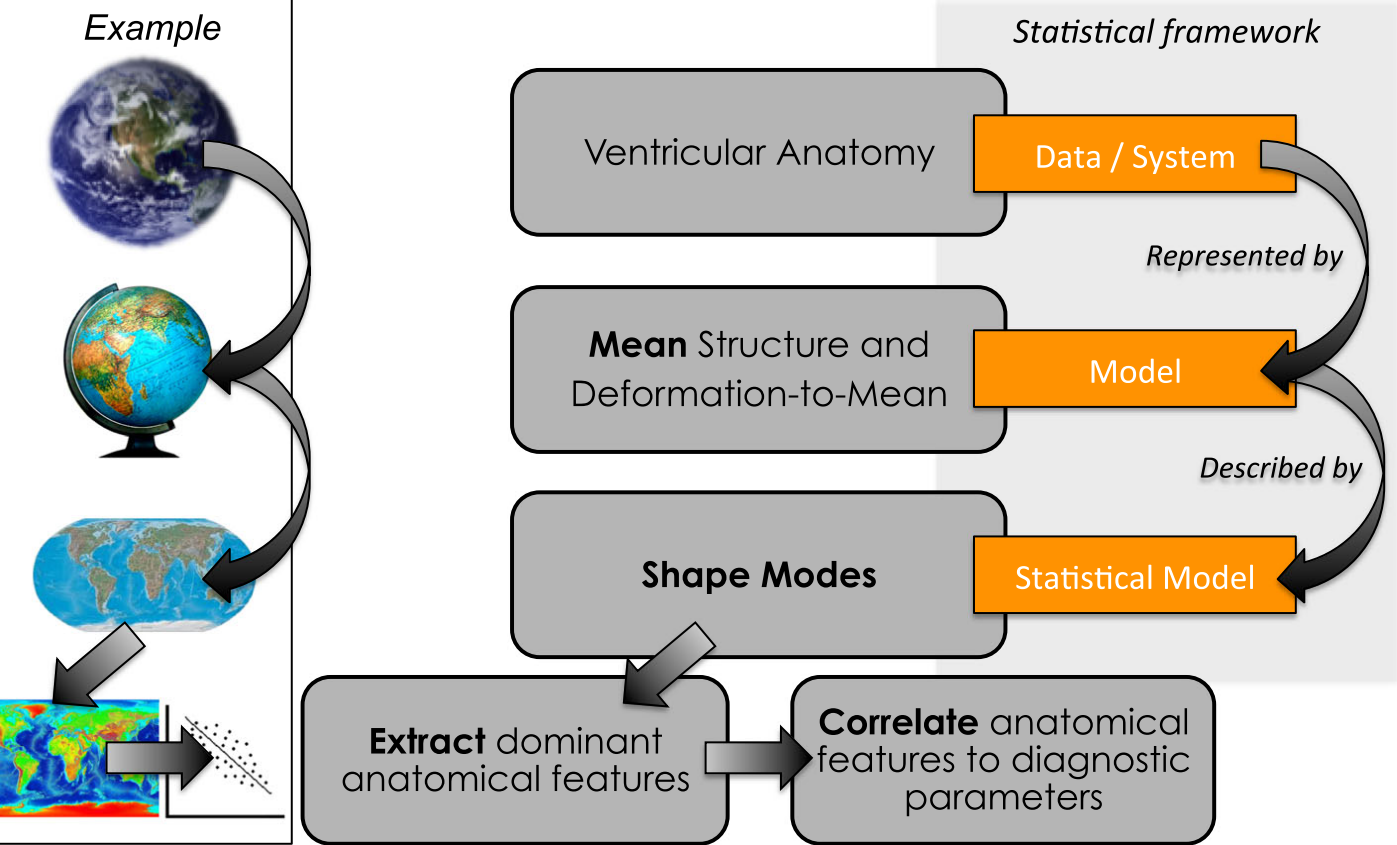
The statistical analysis framework to analyse the anatomical differences between ARVC patients and asymptomatic control subjects by considering the mean (average) anatomy as the model of interest and describing all other subjects as a deformation of this model. The subject-specific deformations can be analysed to extract dominant anatomical features in the population (modes), as well as studying the relationship between the anatomical features and clinical diagnostic parameters. A real-world example is shown by considering the geography of the earth, representing this in 3D with a scale model (a globe), projecting this to 2D (a map), and extracting dominant geographical features such as altitude, to correlate with an external geographical parameter (temperature for example)

The mean (average) and shape modes (dominant shape patterns) in the data were computed from anatomical models that represented the shape of the ventricles (the endocardial and epicardial surfaces) in 3D, following a statistical analysis framework using PCA, as described in detail in [17] and summarised in Fig. 2. The cut-off for the number of shapes was set to the number required to capture 80 % of the shape variance in the population. Further shapes can be computed (up to one less than the number of subjects in the population = 26), however only the most significant, and most widely present shapes in the population were of interest. Subsequent shapes captured little variance in the population and were thus considered to be subject-specific rather than population-wide (i.e. outlier features). Shapes were computed separately for each population.

### Statistical correlation of shape features with clinical diagnostic indices

PCA was applied to each processed data set to extract the dominant shape trends (called shape modes, hereafter referred to as ‘shapes’) in each population (in order of dominance). Using this method, the shape of a given patient is described as a linear combination of these shapes, providing a reduced-order representation of the structure of each patient’s heart. Shapes are visualised at ±1 standard deviation (SD), though the sign is arbitrary in PCA and is not significant of a ‘positive’ or ‘negative’ shape. Two types of analysis were performed for each population; single ventricle analysis of both the RV and LV (to highlight ventricle-specific features), and bi-ventricle analysis (to account for ventricular interaction). In both the control and ARVC populations, five shapes were sufficient for capturing 80 % of the variance in shape observed, both when computing the shapes individually for each ventricle, and when computing combined shape modes (i.e. 5 RV modes, 5 LV endocardium modes, 5 LV epicardium modes and 5 biventricle modes each captured 80 % of the variance).

In order to analyse the relative statistical importance of each ventricle independently, and to determine where in the cardiac cycle shape metrics were more indicative of clinical measures, canonical correlation analysis (CCA) was performed. Using CCA, the correlation between the combination of all of the dominant shape modes in the dataset and the clinical outcomes was computed. This type of analysis was performed to account for the fact that each subject heart is a combination of modes (shapes), and these combinations could interact in the disease state.

Correlations between biventricle shapes and the clinical indices were computed using both non-parametric statistical tests (Kendall’s *τ*_*b*_ and Spearman’s *ρ*), and a parametric statistical test (Pearson’s *r)*. For each clinical index, the correlation was computed individually due to the overlap of the indices (i.e. univariate analysis, as opposed to multivariate analysis). The cut-off for statistical significance was set at *p < 0.05*.

The currents distance [18] was used to compute a global measure of the difference between the subject-specific bi-ventricle shapes and the mean bi-ventricle shape models (ARVC or control). This was performed to test if a simple 1-parameter global bi-ventricle shape metric could differentiate between the two populations. The currents distance is a global measure of the difference between two sets of currents (each representing a structural model of a given subject). Using a leave-one-out protocol, the bi-ventricle mean of the control group was computed from 20 subjects (*N*-1), and the distance to the excluded subject (the *N* ^th^ subject) was computed in the space of currents. Similarly, the distance from the ARVC patients to the control bi-ventricle mean was also computed in the space of currents. In addition to this global measure, a summed regional measure was computed by adding together the shape mode loadings after scaling and normalisation. A similar leave-one-out protocol was applied to this summed regional measure.

To test the predictive power of using just the distance-to-mean global measure or the summed regional measure to distinguish the groups in terms of bi-ventricular shape, a k-nearest-neighbour classifier [19] (with three neighbours) was constructed for each measure. The k-nearest-neighbour classifier finds the closest k (in our case *k* = 3) neighbours with respect to the measure of interest (in our case the distance-to-mean or summed regional measure) and based on the labels of the closest neighbours, a predicted label is assigned, along with the probability of belonging to the predicted class. We used a leave-one-out approach with this algorithm, where for each subject, the classifier was trained with the labeled data of the other 26 subjects, and the trained classifier then used to predict the class of the left-out subject. Based on the predicted labels and probabilities computed from each leave-one-out experiment, ROC curves were generated and the optimal cut-off (i.e. the threshold value above which subjects are classed as AVRC) was chosen by optimising the accuracy, specificity and sensitivity. The Matlab software from Mathworks (http://www.mathworks.com/) (version 2012b) was used for the classification, with built-in functions to perform the k-nearest-neighbour training (using the *ClassificationKNN* function), and the prediction based on this trained classifier (using the *predict* function).

## Results

### Clinical characteristics

Twenty-seven ARVC patients were included (13 (48 %) male, age 38 ± 14 years) as well as 21 controls (12 (57 %) male, age 64 ± 8). Among the ARVC patients, 12 (44 %) had a history of ventricular tachycardia (VT) or ventricular fibrillation (VF), 4 (15 %) patients had survived a cardiac arrest and 8 (30 %) reported previous syncope. Furthermore, 8 (30 %) had an implantable cardioverter defibrillator (ICD) implanted after the CMR examination. Regarding criteria from the 2010 TFC, 12 (44 %) fulfilled major criteria on ECG, 2 had ventricular fat infiltration and 6 had fibrosis by CMR. The number of major and minor criteria according to the TFC varied from 1 to 5 and 0 to 2 respectively (see Table 1 for all clinical diagnostic indices).

**Table 1:** Table of clinical parameters

ARVC diagnosis	TFC major	TFC minor	CMR major	CMR minor	VT/VF	ICD	Cardiac arrest	Syncope	ECG major	SA-ECG	Fat	Fibrosis
1	1	0	0	0	0	0	0	0	0	0	0	1
1	1	0	0	0	0	0	0	1	0	0	0	0
3	2	0	0	0	1	1	1	1	0	0	0	0
3	1	2	0	1	1	0	0	0	0	1	1	1
3	2	2	1	0	0	0	0	0	1	1	0	1
3	2	1	1	0	0	0	0	0	0	0	0	0
3	2	1	1	0	1	0	0	1	1	0	0	0
3	5	1	0	1	1	1	1	1	1	1	0	0
1	1	0	0	0	0	0	0	0	0	0	0	0
1	1	0	0	0	0	0	0	0	0	0	0	0
1	1	0	0	0	0	0	0	0	0	0	0	0
3	2	0	1	0	1	1	1	1	0	0	0	1
2	1	1	0	0	0	0	0	0	0	1	0	0
3	2	1	0	0	1	1	1	1	0	1	0	0
3	4	1	1	0	1	1	0	0	1	1	0	0
3	5	0	1	0	1	1	0	0	1	1	0	1
3	4	1	1	0	1	1	0	0	1	1	0	1
3	2	1	0	0	1	0	0	1	1	0	0	0
3	5	0	1	0	1	1	0	1	1	1	0	0
3	2	0	0	0	0	0	0	0	1	0	0	0
3	1	2	0	0	0	0	0	0	1	0	0	0
1	1	0	0	0	0	0	0	0	0	0	0	0
1	1	0	0	0	0	0	0	0	0	1	0	0
1	1	0	0	0	0	0	0	0	0	0	0	0
3	3	1	1	0	1	0	0	0	1	0	0	0
3	2	0	1	0	0	0	0	0	1	0	0	0
2	1	1	1	0	0	0	0	0	0	1	1	0

Clinical findings and diagnostic indices from the 2010 TFC [2] for the 27 ARVC patients. Patients reported as having an ICD were scanned prior to implantation. ARVC diagnosis: 3 = definite, 2 = borderline, 1 = possible

*Major* number of TFC major criteria, *Minor* number of TFC minor criteria, *VT/VF* history of ventricular arrhythmias, *ICD* implantable cardioverter defibrillator, *ECG major* electrocardiogram major TFC criteria, *SA-ECG* TFC from signal-averaged electrocardiogram

### Single ventricle analysis

**Fig. 3:**
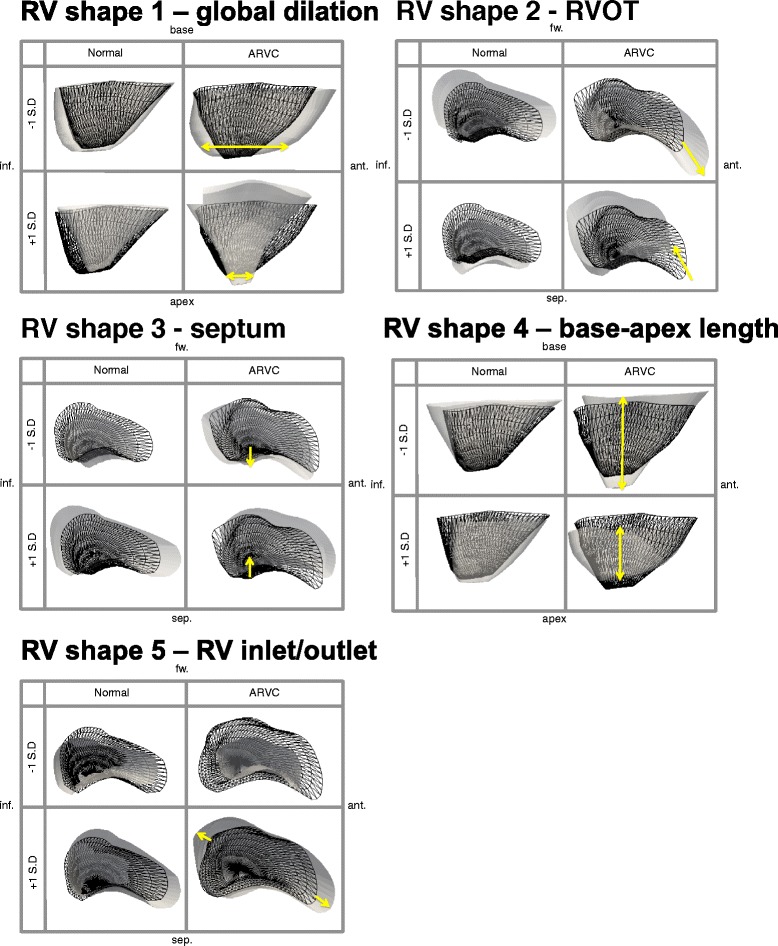
The first five RV shapes, in descending order from most prominent in the population to least prominent, for the control group (*left*) and the ARVC group (*right*) shown at ±1 standard deviation (SD) at the ES phase. In each box, the mean for the group (control or ARVC) is shown as reference in black wireframe, and the shape at each extreme (±1 SD) is shown in solid white. The yellow arrows indicate the location and direction of greatest shape variance in the ARVC shapes, which showed global dilation/shrinking for shape 1, elongation/shortening at the RV outflow tract for shape 2, titling (leaning) from apex to base at the septum for shape 3, lengthening/shortening for shape 4 and bulging/shortening at both the RV inlet and RV outlet for shape 5. These shapes are consistent with known shape abnormalities in these patients. Note that the sign (+/-) is not indicative of positive/negative change in PCA and is thus arbitrarily assigned. Abbreviations: inf: inferior, sep: septal, ant: anterior, fw: RV free wall

**Fig. 4:**
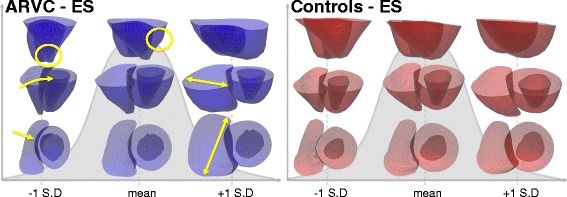
The mean and first PCA shape (shown at ±1SD) for the ARVC group (*left*) and the control group (*right*) at the end systolic (ES) frame. The first shapes capture the size variance in each population, since no size correction was performed prior to analysis. The yellow arrows at -1SD highlight the narrow RV that is tilted towards the LV, the yellow circle at -1SD highlights the narrow apex, the yellow circle at the mean shows a bulge in the RV, and the yellow double sided arrows at +1SD highlight the dilation of the RV. Note that the top row is showing the RV in front of the LV

The RV shape features (shown in Fig. 3) have visibly identifiable differences. This was in contrast to the LV shapes, which showed few visible distinguishing features. The first five RV shapes at ±1 standard deviation (SD) are shown in Fig. 3 from different views to emphasise the largest shape difference observed for each (in terms of differences from the control group mean). Since similar shapes were found at ED and ES for the RV shapes, only the ES shapes are shown (ES was slightly more discriminant, see Table 2). The shapes were ordered according to how commonly they were present in this population (from most to least common). The first ARVC RV shape, which explained 44 % of the shape variance in the population, appeared to show dilation/shrinking, particularly around the apical region. The second RV shape explained 15 % of the variance and showed elongation/shortening at the RV outflow tract. The third RV shape, which explained 10 % of the variance, showed a tilting from the apex to the base at the septum (i.e. RV septum tilting inwards/outwards at the base with respect to the location of the apex). The fourth RV shape, which explained 7 % of the variance, showed lengthening/shortening in the RV for this group. Finally, the fifth RV shape, which explained 5 % of the variance, showed abnormal bulging/shrinking at both the RV inlet and outlet (in contrast to RV shape 2 which dilates at +1SD, but not in combination to the RVOT elongation). Meanwhile, labelling of the LV shapes according to what they anatomically represent was not possible given the small amount of visually distinguishable shape differences between the first LV shape and the other LV shapes. The first shapes computed for the LV alone in this section of the analysis were visually similar to the first LV shapes computed from the bi-ventricle analysis for each population (described in the following section) and are shown in Fig. 4.

**Table 2:** Table of correlations

Clinical Indices	LV endocardium		LV epicardium		RV endocardium	
	ED phase	ES phase	ED phase	ES phase	ED phase	ES phase
BSA	*p = 0.46*	*p = 0.17*	*p = 0.06*	*p = 0.05*	*p = 0.32*	*p = 0.12*
Gender	*p = 0.20*	***p = 0.03***	***p = 0.05***	***p = 0.03***	*p = 0.22*	***p = 0.08***
No. major	*p = 0.24*	***p = 0.02***	*p = 0.14*	***p = 0.005***	***p = 0.04***	***p = 0.004***
No. minor	*p = 0.57*	*p = 0.94*	*p = 0.81*	*p = 0.94*	*p = 1.00*	*p = 0.88*
VT/VF	*p = 0.17*	*p = 0.18*	***p = 0.05***	*p = 0.12*	***p = 0.04***	***p = 0.03***
Cardiac arrest	*p = 0.43*	*p = 0.63*	***p = 0.02***	*p = 0.13*	*p = 0.07*	***p = 0.04***
Syncope	*p = 0.78*	*p = 0.41*	*p = 0.26*	*p = 0.77*	*p = 0.90*	*p = 0.57*
ECG major	*p = 0.18*	*p = 0.17*	*p = 0.21*	*p = 0.15*	***p = 0.02***	***p = 0.04***
SAECG	*p = 0.12*	*p = 0.22*	*p = 0.07*	*p = 0.11*	*p = 0.19*	***p = 0.003***
Fat	*p = 0.21*	*p = 0.49*	*p = 0.19*	*p = 0.22*	*p = 0.46*	*p = 0.61*
Fibrosis	***p = 0.04***	*p = 0.31*	*p = 0.12*	*p = 0.083*	***p = 0.04***	*p = 0.33*

Table of canonical correlations between the first 5 shape modes, and each of the clinical indices (significant correlations with *p < 0.05* shown in bold). The individual RV shapes are shown in Fig. 3. The individual LV shapes are not shown, though an example of the first LV shape can be see in Fig. 4 from the bi-ventricle analysis

*LV* left ventricle, *RV* right ventricle, *BSA* body surface area, *No. major* number of major task force criteria (TFC), *No. minor* number of minor TFC, *VT/VF* history of ventricular arrhythmias, *ECG major* major TFC from electrocardiogram, *SAECG* major TFC from signal-averaged ECG, *ED* end-diastole, *ES* end-systole

The canonical correlations between the combination of the first five shapes for each ventricle individually, and separately for the LV endocardium and LV epicardium (representing >80 % of the variance in the population in all configurations) and clinical parameters, computed using CCA, are given in Table 2. Common correlations were found for the LV and the RV for the number of major criteria, history of arrhythmias, history of cardiac arrest and presence of fibrosis. Additional correlations were found in the RV for ECG major criteria and signal-averaged ECG, and in the LV for gender.

### Bi-ventricular analysis

The mean structure and most dominant shape features for each population were computed for the bi-ventricle models at ED and ES. Similar results were found at both phases for all shapes and the results at ES for each group for the first shape at ±1 SD are shown in Fig. 4. At -1 SD, some differences between the ARVC group and the control group at the septum are visible, as shown with yellow arrows in the middle and bottom rows. For the remaining shapes, the RV part of the bi-ventricle shapes were visually similar to those computed individually for the RV in Fig. 3 (and are thus not shown again), and as in the single-ventricle analysis of the LV, little LV shape differences were visually distinguishable.

**Fig. 5:**
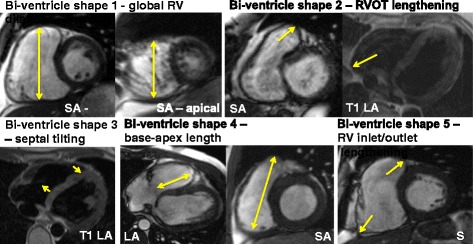
CMR of the patients with the largest shape loadings for each shape. The short axis (SA) views around the basal and apical regions showed global dilation in patient 26, the SA and T1-weighted long axis (LA) images for patient 24 displayed the elongation at the RV outflow tract (RVOT), the T1-weighted LA image of patient 3 showed the tilting of the RV from the apex to the base at the septum, the LA view showed shortening of the RV (shown in the SA view) for patient 4, and the SA view of patient 16 showed dilation at the RV inlet and outlet. Note that the T1 images shown here are for visualisation purposes only, but were not used as a part of this study

In order to highlight the identified individual shape features that make up the ARVC population, CMR images of the patients with the largest bi-ventricle shape loadings are shown in Fig. 5. Using these outliers, it is possible to visualise the abnormalities on the images directly. Note that T1-weighted images are shown for visualisation purposes only and were not used for the analysis.

**Fig. 6:**
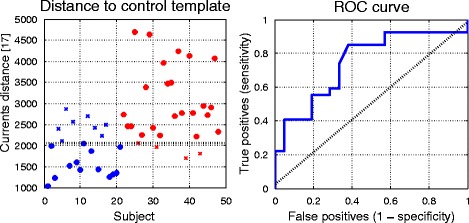
*Left*: The currents distances (see [17] for a full description of this distance measure) plotted for all subjects with controls shown in blue, ARVC shown in red. Crosses denote misclassified subjects (i.e. controls misclassified as ARVC, ARVC misclassified as controls) from the classifier trained on the currents distance measures in a leave-one-out protocol. The line that divides the predicted groups is shown in black. *Right*: The ROC curve from the classification (blue line) and predictive accuracy that would result from random guess (dashed diagonal black line)

Using the global measure of currents computed from the bi-ventricle mean, the predictive accuracy from the trained classifier (trained from the currents distance measures only) was 75 %, with 85 % accuracy of prediction for the ARVC subjects (i.e. high sensitivity) and 62 % accuracy for the control group (i.e. low specificity). The currents distances are plotted for each subject in Fig. 6 (left), for the control group (coloured in blue) and the ARVC group (plotted in red). The subjects that were misclassified with the trained classifier are indicated with crosses. The receiver operating characteristic (ROC) curve summarising the performance of the classifier is shown in Fig. 6 (right). Using a single measure of distance amounts to quantifying global bi-ventricle differences and thus neglects more localised abnormalities such as bulging or elongation.

**Fig. 7:**
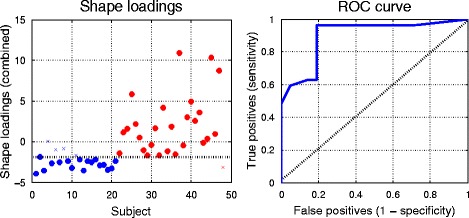
*Left*: The sum of the shape loadings (after normalisation and scaling) for all subjects with controls shown in blue, ARVC shown in red. Crosses denote misclassified subjects (i.e. controls misclassified as ARVC, ARVC misclassified as controls) from the classifier trained on the sum of the loadings using a leave-one-out protocol. Note that the misclassified ARVC patient was a patient with a borderline diagnosis of ARVC. The line that divides the predicted groups is shown in black. *Right*: The ROC curve from the classification (*blue line*) and predictive accuracy that would result from random guess (dashed diagonal black line)

The same classifier trained similarly on the summed bi-ventricle shape loadings from the regional bi-ventricle shape descriptors gave a predictive accuracy of 90 %, with 96 % accuracy of prediction for the ARVC subjects (i.e. high sensitivity) and 81 % accuracy for the control group (i.e. moderate specificity). The summed shape loadings (after normalisation and scaling) are plotted for each subject in Fig. 7 (left), for the control group (coloured in blue) and the ARVC group (plotted in red). The subjects that were misclassified with the trained classifier are indicated with crosses. The only misclassified ARVC patient was one with a borderline diagnosis of ARVC. The receiver operating characteristic (ROC) curve summarising the performance of the classifier is shown in Fig. 7 (right).

**Fig. 8:**
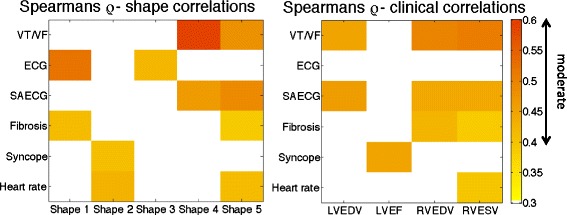
Absolute *ρ* values computed using Spearman’s non-parametric statistical test plotted for the bi-ventricle shapes showing only the statistically significant (*p* < 0.05) values. Correlations with additional standard clinical indices, computed from the Segment software [16], are also shown: LVEDV/RVEDV: left ventricle (LV)/right ventricle (RV) end-diastolic volume, RVESV: RV end- systolic volume; LVEF: LV ejection fraction

The statistically significant (*p < 0.05*) *ρ* values from Spearman’s rank correlation coefficient test are shown in Fig. 8 between the clinical diagnostic indices and each bi-ventricle shape (left) and with clinical metrics computed automatically from the Segment software, shown for comparison (right), showing only the absolute values of *ρ* (since degree of correlation and not direction is of interest in this study). Similar results were found for Kendall’s rank correlation coefficient and Pearson’s product-moment correlation coefficient. Note that given the small population size and exploratory nature of this study, correction for multiple correlations was not performed.

**Fig. 9:**
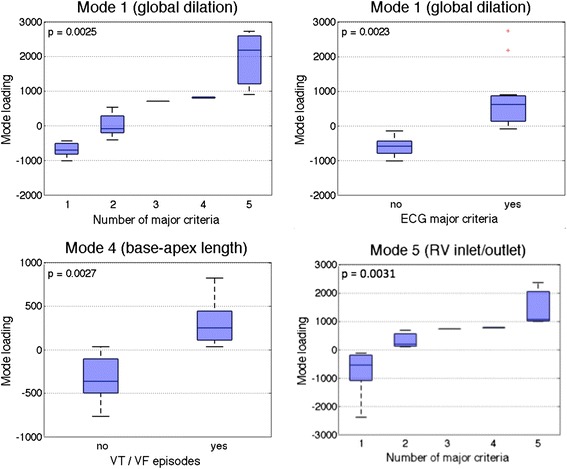
Loadings of bi-ventricle shape 1 plotted against the number of major TFC criteria (*top left*), ECG major criteria (*top right*), bi-ventricle shape 4 plotted against history of arrhythmias (*bottom left*), and bi-ventricle shape 5 plotted against the number of major TFC criteria (*bottom right*). The loadings represent how present the shape is in each patient. Note that shape 1 represented global dilation of the RV, shape 4 represented base-apex length of the RV, and shape 5 represented bulging at the inlet and outlet of the RV (see Fig. 5)

The bi-ventricle shape loadings are plotted against the clinical diagnostic indices in Fig. 9 for the most statistically significant shape/index combinations. The shape loadings represent how present each shape was in each patient (e.g. how elongated the RVOT was from shape 2). Clear trends were visible, where larger loadings of shape 1 appear to suggest more major criteria, and similarly for shape 5. Patients with ECG major criteria had larger loadings of shape 1. Larger loadings of shape 4 and 5 appear to suggest higher rates of arrhythmias.

## Discussion

In this study, using computational methods, CMR data from ARVC patients and controls was summarized into models of ventricular structure dominated by 5 particular ventricular shapes features for both the single ventricle (i.e. 5 RV shapes, 5 LV endocardium shapes, 5 LV epicardium shapes) and bi-ventricle analyses (5 combined LV/RV shapes). This represents a novel approach to ARVC imaging by using true 3D structure of the ventricles in contrast to traditional measures of function and simple chamber diameters. The dominant RV shapes in the ARVC population were global RV dilation/shrinking, elongation/shortening at the RVOT, tilting at the septum, shortening/lengthening of the ventricles and bulging/shortening at the RV inlet and outlet. All these are known ARVC structural features, which with the presented tool can now be quantitatively measured in new patients. Our study also related the importance of the different shape features to ARVC patients’ clinical findings and outcome. We suggest that this image shape analysis approach in ARVC may help understanding of ARVC development, may help monitor disease progression, and potentially help risk stratification of ventricular arrhythmias.

### Frequency of different shapes and clinical relationships

The most common RV shape (shape 1) was characterized by RV dilation and explained 44 % of the shape variance in the population. RV dilation is known as an early sign of ARVC [15]. Shape 1 correlated to ECG criteria, which are also known to be an early sign of ARVC [11]. Interestingly, bi-ventricle shape 1 (which also represented RV dilation) was also correlated to fibrosis by CMR. Shortening/lengthening of the ventricles (shape 4) and bulging/shortening at the RV inlet and outlet (shape 5) were less common in the ARVC population, but were more highly correlated with adverse clinical outcomes such as ventricular arrhythmias. The correlations between the bi-ventricle shape modes and clinical symptoms were consistent with expected relationships between shape abnormalities and ECG major criteria, signal-averaged ECG, history of arrhythmias, syncope, as well as presence of fibrosis on CMR. Interestingly, while volume measures were correlated to VT/VF, the first shape was not correlated, despite the fact that this shape captured the dilation of both ventricles. This suggests that the shape may contain other features not related to dilation that are not directly visible.

We correlated the shapes to clinical parameters included in the TFC, and to clinical outcome such as ventricular arrhythmias. Due to the small number of included patients in this study, the correlations to clinical parameters were intended as a pilot study and should be interpreted with care. However, these pilot results indicate that studying the relationship between symptoms and the shape modes extracted from population-based analysis could provide insight into which shape modes may be indicative of symptomatic ARVC. Therefore, future studies should explore if the assessment of shape can predict ventricular arrhythmias when new patients present large loadings of shape 4 and 5. Furthermore, patients presenting with lower loadings of shape 4 and 5 would potentially be at lower risk of events. Such analysis could thus be used to determine patient risk of future events, as a secondary means of support for existing clinical metrics. Given the exploratory nature of the present study where no specific hypotheses were pre-specified and where the objective was to identify important shape features and their relationship with clinical outcomes, no correction for multiple testing was performed. With the number of comparisons made here, this increases the likelihood of making a Type I error (i.e. identifying an effect that doesn’t exist). However, for this study, these are preferable to Type II errors (i.e. missing an effect when one existed) given the potentially lethal nature of this condition.

Correlating shape with clinical indices was addressed in a recent study of regional structural abnormalities in mutation-positive ARVC patients using a manual, qualitative identification of abnormal regions [11], in contrast to the automatic methods proposed in this work. While statistically significant correlations were found between qualitatively defined structural severity and ECG findings (consistent with our findings), there was no significant correlation for SAECG or syncope (in contrast to our findings) from the qualitative shape descriptors.

### Measurement of single versus bi ventricular shapes

The shape modes represent the most commonly occurring shape features in the population and can be used to study abnormalities at a population-level. In this study, both single-ventricle models and a combined bi-ventricular model were built from CMR images. The single-ventricle models treat the shape abnormalities for only a given ventricle and neglect the ventricular interaction. Interestingly, despite the fact that there were no identifiable shape abnormalities visible in the computed LV shapes (both when computing the LV shapes separately, and when computing the combined bi-ventricle shapes), the LV shapes were correlated to clinical indices. Furthermore, differences between the endocardium and epicardium were found. Due to the size of the population in this study, few patients had severe ARVC with LV involvement, therefore making it difficult to identify population-based LV features. In a larger study these features may become more apparent and may lead to LV shapes that are more qualitatively informative. Since LV involvement is an indication of severe ARVC it is not surprising that LV shapes could be related to adverse outcomes, though it is difficult to conclude this based on the present study, and furthermore, it is difficult to conclude why the LV epicardium shapes were more correlated than the LV endocardium shapes.

Meanwhile, the bi-ventricular model can account for the inherent coupling between the ventricles. The advantage of the single-ventricle models is that discovered shape features are easier to interpret, and the correlations between each ventricle and the clinical diagnostic indices can be analysed independently. The bi-ventricular model, on the other hand, is able to maintain the ventricular interaction, but at the expense of more complicated shape features being calculated. Statistical analysis was performed by both analysing the correlation between individual shapes, and by studying the relationship between clinical indices and the combination of the most dominant shapes. Studying the shapes individually is important to identify how each shape individually relates to clinical features. Studying the combination of shapes is important, as each patient is a combination of shapes that may have a high degree of interaction. Therefore, by studying the relationship between the combination of shapes and clinical indices we can understand how the total shape (combination of shape modes) is related to clinical features, and how each shape individually contributes to that relationship (as computed using CCA). Both approaches hold clinical merit for patient risk stratification.

The global bi-ventricle distance classification yielded high sensitivity (i.e. 85 % of ARVC patients were correctly identified as ARVC), but low specificity (i.e. 62 % of control subjects were correctly classified). The classification from the bi-ventricle shape loadings yielded higher accuracy, with high sensitivity (96 %) and moderate specificity (81 %). For ARVC diagnosis, high sensitivity is more important than high specificity due to the potentially life-threatening outcomes of this disease. The classification accuracy from the shape loadings was found to be very promising given that shape information alone was used to classify the subjects. Thus the global measure used in conjunction with regional shape analysis could have potential use for clinical secondary diagnostic support.

### Clinical implications

The clinical implications of a shape-based imaging approach in ARVC patients needs to be further explored. Our clinical investigation was considered as a feasibility study, showing the potential of this method for ARVC diagnosing, monitoring of disease progression and risk stratification for life-threatening events. In future studies, shape loadings could be computed for new subjects by computing the deformation from the subject to the mean and projecting the deformation for each shape, which could thus quantify shape abnormalities in new patients. In addition, future prospective studies should assess in a larger population if this type of analysis can be used to predict and risk-stratify patients based on 3D descriptors of ventricular structure, directly following clinical manifestations over time. Furthermore, the current study relied on image segmentation tools that are not currently fully automated. Since the analysis of the quality of the image segmentation was not the focus of this study, a single observer performed all image processing tasks for consistency, but no test/retest variability for the image segmentation was performed. All other aspects of the proposed methods are unbiased, and when automatic tools for segmenting the structure from CMR become available, the proposed measures will be fully unbiased.

### Limitations

Structural analysis in this work relied on extracting the 3D structures of interest from CMR, which is a challenging task. In this study we used a combination of manual and automated approaches. Manual delineation of a region of interest can be more straightforward than automatic methods, however, manual methods can be time consuming and subject to user-bias. Several methods for automatically or semi-automatically extracting (segmenting) the ventricles from cardiac images have been proposed (see [20] for a review of recent methods). The current analysis used such techniques to create 3D models that extended up to the base and were cut flat at the slice below the first valve insertion visible in either the LV or RV. Including the valves would provide further details for the structure, particularly at the RVOT. This limitation is due to the challenge of extracting 3D models that extend as far as the valves. Methods for this are currently being developed, and when these become available, analysis of the full ventricle will be performed.

Computing the shape modes in a population can be performed either with a non-supervised method or with a supervised method. In terms of 3D shape, there can be several (even an infinite number) modes, in contrast to the mode of a set of numbers, which has a single unique value. The method used in this work was PCA, which is a popular non-supervised projection method and is convenient for extracting the most dominating features in a dataset [8]. Supervised methods may provide more insight into which shape features are related to specific indices, and can be performed in future work knowing which indices are relevant to study, as determined from the present analysis.

The control group used in this study came from an ageing population to establish the difference (if one existed) between age-related remodelling and disease-related remodelling. However, it would also be of interest to compare an age-matched healthy control group to the ARVC group to distinguish and compare healthy and ARVC-related shape features. Additionally, the control subjects used in this work were obtained from a dataset of patients with suspected ischemic cardiac disease and are hence not necessarily healthy control subjects. Thus, some of the control subjects may have abnormal shape features related to non-cardiac conditions. If this is the case, the results in this work suggest that the proposed methods are able to distinguish remodelling due to ageing and other conditions from ARVC remodelling.

## Conclusions

This study showed for the first time a novel quantitative approach for assessing ARVC disease beyond state-of-the-art measures by quantifying ventricular shape abnormalities in ARVC patients using computational methods. This work demonstrated the ability to use advanced shape analysis tools to extract shape features from CMR. We summarized the 5 most frequent ventricular shapes in ARVC patients and related these shapes to clinical manifestations. Our findings indicate the potential of using 3D shape analysis as support for future clinical decision-making by providing additional indicators of disease staging and arrhythmia risk.

## Additional file

Additional file 1: File containing the supplementary material. (PDF 550 kb)

## Abbreviations

3D: Three-dimensional
4D: Four-dimensional (3D + time)
ARVC: Arrhythmogenic right ventricular cardiomyopathy/dysplasia
CCA: Canonical correlation analysis
CMR: Cardiovascular magnetic resonance
ECG: Electrocardiography
ED: End-diastolic phase
ES: End-systolic phase
ICD: Implantable cardioverter-defibrillator
LV: Left ventricle
PCA: Principal Component analysis
ROC: Receiver operating characteristic
RV: Right ventricle
SAECG: Signal-averaged electrocardiography
TFC: Task force criteria

## References

[1] Corrado D, Basso C, Thiene G, McKenna WJ, Davies MJ, Fontaliran F, Nava A, Silvestri F, Blomstrom-Lundqvist C, Wlodarska EK (1997). Spectrum of clinicopathologic manifestations of arrhythmogenic right ventricular cardiomyopathy/dysplasia: a multicenter study. J Am Coll Cardiol.

[2] Marcus FI, McKenna WJ, Sherrill D, Basso C, Bauce B, Bluemke DA, Calkins H, Corrado D, Cox MG, Daubert JP (2010). Diagnosis of arrhythmogenic right ventricular cardiomyopathy/dysplasia. Eur Heart J.

[3] Bluemke DA, Krupinski EA, Ovitt T, Gear K, Unger E, Axel L, Boxt LM, Casolo G, Ferrari VA, Funaki B (2003). MR imaging of arrhythmogenic right ventricular cardiomyopathy: morphologic findings and interobserver reliability. Cardiology.

[4] Tandri H, Saranathan M, Rodriguez ER, Martinez C, Bomma C, Nasir K, Rosen B, Lima JA, Calkins H, Bluemke DA (2005). Noninvasive detection of myocardial fibrosis in arrhythmogenic right ventricular cardiomyopathy using delayed-enhancement magnetic resonance imaging. J Am Coll Cardiol.

[5] Marcus F, Fontaine G, Guiraudon G, Frank R, Laurenceau J, Malergue C, Grosgogeat Y (1982). Right ventricular dysplasia: a report of 24 adult cases. Circulation.

[6] Lang RM, Badano LP, Mor-Avi V, Afilalo J, Armstrong A, Ernande L, Flachskampf FA, Foster E, Goldstein SA, Kuznetsova T, Lancellotti P (2015). Recommendations for cardiac chamber quantification by echocardiography in adults: an update from the American Society of Echocardiography and the European Association of Cardiovascular Imaging. J Am Soc Echocardiogr.

[7] Medrano-Gracia P, Cowan BR, Ambale-Venkatesh B, Bluemke DA, Eng J, Finn JP, Fonseca CG, Lima J, Suinesiaputra A, Young AA (2014). Left ventricular shape variation in asymptomatic populations: the multi-ethnic study of atherosclerosis. J Cardiovasc Magn Reson.

[8] Wold S, Esbensen K, Geladi P (1987). Principal component analysis. Chemom Intel Lab Syst.

[9] Mansi T, Voigt I, Leonardi B, Pennec X, Durrleman S, Sermesant M, Delingette H, Taylor AM, Boudjemline Y, Pongiglione G (2011). A statistical model for quantification and prediction of cardiac remodelling: Application to tetralogy of fallot. IEEE Trans Med Imaging.

[10] Corrado D, Wichter T, Link MS, Hauer R, Marchlinski F, Anastasakis A, Bauce B, Basso C, Brunckhorst C, Tsatsopoulou A (2015). Treatment of arrhythmogenic right ventricular cardiomyopathy/dysplasia: an international task force consensus statement. Eur Heart J.

[11] Te Riele AS, James CA, Philips B, Rastegar N, Bhonsale A, Groeneweg JA, Murray B, Tichnell C, Judge DP, HEIJDEN JF (2013). Mutation-positive arrhythmogenic right ventricular dysplasia/cardiomyopathy: The triangle of dysplasia displaced. J Cardiovasc Electrophysiol.

[12] Saberniak J, Hasselberg N, Sarvari S, Ribe M, Borgquist R, Platonov P, Smith H-J, Edvardsen T, Haugaa K (2014). Exercise impairs myocardial function assessed by magnetic resonance imaging in subjects with arrhythmogenic right ventricular cardiomyopathy. J Am Coll Cardiol.

[13] Sarvari SI, Haugaa KH, Anfinsen O-G, Leren TP, Smiseth OA, Kongsgaard E, Amlie JP, Edvardsen T (2011). Right ventricular mechanical dispersion is related to malignant arrhythmias: a study of patients with arrhythmogenic right ventricular cardiomyopathy and subclinical right ventricular dysfunction. Eur Heart J.

[14] McLeod K, Mansi T, Sermesant M, Pongiglione G, Pennec X. Statistical shape analysis of surfaces in medical images applied to the Tetralogy of Fallot heart. In: Modeling in Computational Biology and Biomedicine. Springer Berlin Heidelberg; 2013. p. 165-191.

[15] Saberniak J, Leren IS, Haland TF, Beitnes JO, Hopp E, Borgquist R, Edvardsen T, Haugaa KH. Comparison of patients with early-phase arrhythmogenic right ventricular cardiomyopathy and right ventricular outflow tract ventricular tachycardia. Eur Heart J Cardiovasc Imaging. 2016;14. [Epub ahead of print]. 10.1093/ehjci/jew014 PMC5217739 26903598

[16] Heiberg E, Sjögren J, Ugander M, Carlsson M, Engblom H, Arheden H (2010). Design and validation of segment-freely available software for cardiovascular image analysis. BMC Med Imaging.

[17] Bruse JL, McLeod K, Biglino G, Ntsinjana HN, Capelli C, Hsia TY, Sermesant M, Pennec X, Taylor AM, Schievano S (2016). A statistical shape modelling framework to extract 3D shape biomarkers from medical imaging data: assessing arch morphology of repaired coarctation of the aorta. BMC Med Imaging.

[18] Durrleman S, Pennec X, Trouvé A, Ayache N (2009). Statistical models of sets of curves and surfaces based on currents. Med Image Anal.

[19] Cover T, Hart P (1967). Nearest neighbor pattern classification. IEEE Trans Inf Theory.

[20] Petitjean C, Dacher J-N (2011). A review of segmentation methods in short axis cardiac MR images. Med Image Anal.

