# Supplementary material for: Ventricular structure in ARVC: going beyond volumes as a measure of risk
Source: J Cardiovasc Magn Reson. 2016 Oct 14;18:73. doi: 10.1186/s12968-016-0291-9 (PMC5069945; doi:10.1186/s12968-016-0291-9)
Supplement: Supplementary file 1 — File containing the supplementary material. (PDF 550 kb) [file 12968_2016_291_MOESM1_ESM.pdf]

Supplementary Material

From CMR Sequences to 4D anatomical models

In the present study, an open source segmentation software, Segment from MedViso [1], was used to perform four-dimensional (4D) segmentation by applying the automatic tools, and refining the results as needed. From the Segment software, point clouds of points defining the endocardial (or epicardial) borders were saved for each image slice of the cine stack. These point clouds were processed directly to remove slice misalignment (due to breath-hold misalignment in the CMR images). Slice misalignment correction was carried out by computing the barycentre of each set of points (for each slice) and using linear regression to align the barycentre of a given slice to the barycentre of the slice above and the slice below. The pre-processing steps are summarised in Fig. 1, and described further in [2].

Spatial and temporal alignment of anatomical models

In order to quantitatively compare the anatomical models of different subjects, the models were first aligned in space and the correct temporal frames were extracted. Spatial alignment reduced the bias in the construction of the mean (described in the next section) to allow differences in the anatomy to be computed rather than differences in position and orientation. Temporal alignment ensured that corresponding time frames were compared. The spatial alignment was performed on the extracted point clouds, rather than on the images themselves, given that points are simpler to align than images.

The spatial alignment used in this work was rigid; essentially aligning all subjects to the same physical space without any stretching/shortening or scaling. In order to align the different subjects, the barycentre of each ventricle over all slices was computed and a line-segment joining the barycentre of the left ventricle (LV) to the barycentre of the right ventricle (RV) was computed. The line-segments were then aligned pair-wise to a chosen reference subject for all other subjects via rotation and translation operations. The computed line-segment transformation was then applied to all points. Note that the choice of reference subject was arbitrary in this formulation.

The temporal alignment used in this work was designed to account for the fact that CMR sequences are gated by the electrocardiogram (ECG) signal, while taking into consideration the different heart rates and ES phase from one subject to another. Assuming the ECG-gating was accurate, the first and last frames of the CMR sequence should correspond to the ED phase. Thus, we chose the first frame as the ED phase. The ES frame was taken from the LV volume curves computed from Segment for each subject by choosing the frame that gave the minimum volume.

Once all sets of point clouds were aligned to a common spatial frame and corresponding temporal frames were extracted, an anatomical surface model was created from each point cloud using the Gmsh software [3], and smoothed to obtain a more physiological shape using the Visualisation Toolkit (VTK) [4].

Mean model construction

In the present work, we were interested in computing a mean shape model without a parameterisation of the surfaces (namely without defining any landmarks, and

point-correspondences from one anatomical model to another). A non-parametric (no point labelling) method was used to describe each anatomical model as a set of 'currents', from which statistical analysis was performed on the set of currents rather than on the anatomical models themselves. The currents representation of shapes essentially describes a distribution of shape features by characterising the surfaces by how they 'interact' with vector fields; which is a mathematical concept that can be considered intuitively as the same action that a laser scanner has to describe the shape of a 3D object by moving a laser over the object. Describing the shapes (surfaces) in this way has the advantage of removing the parameterisation of points while providing a framework where two shapes can be quantitatively compared.

A forward model was then used to describe each subject shape by how the mean shape was stretched/shortened to obtain this shape (the deformation required to transform shape A to shape B), plus some residual information not included in the analysis (such as errors/bias due to image quality, segmentation, etc.). Using this formulation, the mean was computed iteratively by minimising the 'distance' between the mean and all subjects (or more precisely, by minimising the deformations from each subject to the mean). The deformations were computed using the large deformation diffeomorphic metric mapping method (LDDMM). The full mean shape model construction pipeline is described in [5], the application of this method for right ventricular shape analysis is described in [6] and the extension of this to bi-ventricular analysis is described in [7].

Author details

References

1. Heiberg, E., Sjögren, J., Ugander, M., Carlsson, M., Engblom, H., Arheden, H.: Design and validation of segment-freely available software for cardiovascular image analysis. BMC Medical Imaging **10**(1), 1 (2010)
2. Marciniak, M., Arevalo, H., Tfelt-Hansen, J., Jespersen, T., Jabbari, R., Glinge, C., Vejlstrup, N., Engstrom, T., Maleckar, M.M., McLeod, K.: From mr image to patient-specific simulation and population-based analysis: Tutorial for an openly available image-processing pipeline. Proc. Statistical Atlases and Computational Models of the Heart Workshop, Athens (2016)
3. Geuzaine, C., Remacle, J.-F.: Gmsh: A 3-D finite element mesh generator with built-in pre-and post-processing facilities. International Journal for Numerical Methods in Engineering **79**(11), 1309–1331 (2009)
4. Schroeder, W., Martin, K., Lorensen, B.: The visualization toolkit: an object oriented approach to 3D graphics. New York: Kitware. Inc. Publisher **156** (2003)
5. Durrleman, S., Pennec, X., Trouvé, A., Ayache, N.: Statistical models of sets of curves and surfaces based on currents. Medical Image Analysis **13**(5), 793–808 (2009)
6. Mansi, T., Voigt, I., Leonardi, B., Pennec, X., Durrleman, S., Sermesant, M., Delingette, H., Taylor, A.M., Boudjemline, Y., Pongiglione, G., *et al.*: A statistical model for quantification and prediction of cardiac remodelling: Application to tetralogy of fallot. Medical Imaging, IEEE Transactions on **30**(9), 1605–1616 (2011)
7. McLeod, K., Mansi, T., Sermesant, M., Pongiglione, G., Pennec, X.: Statistical shape analysis of surfaces in medical images applied to the tetralogy of fallot heart. In: Modeling in Computational Biology and Biomedicine, pp. 165–191. Springer, Berlin Heidelberg (2013)

Figures

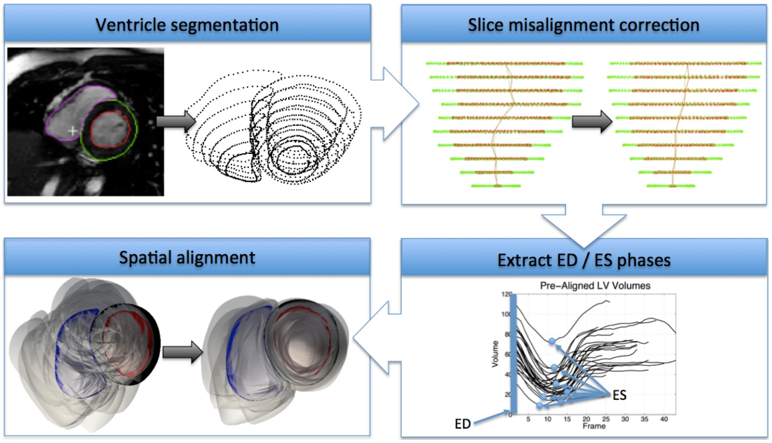

Figure 1 The pre-processing pipeline to go from a CMR image to a point cloud describing the surfaces of the endocardium and epicardium of the left ventricle and endocardium of the right ventricle (*ventricle segmentation*), then to correct the slice misalignment due to breath-holds (*slice misalignment correction*), then to detect the frames corresponding to the ED and ES phases (*extract ED / ES phases*), and finally to align all subjects spatially to remove differences in position and orientation (*spatial alignment*).
